# Reducing meat consumption in Central Asia through 3D printing of plant-based protein—enhanced alternatives—a mini review

**DOI:** 10.3389/fnut.2023.1308836

**Published:** 2024-01-17

**Authors:** Ulanbek Auyeskhan, Arman Azhbagambetov, Temirlan Sadykov, Damira Dairabayeva, Didier Talamona, Mei-Yen Chan

**Affiliations:** ^1^Department of Mechanical & Aerospace Engineering, Nazarbayev University, Astana, Kazakhstan; ^2^Department of Intelligent Systems & Cybersecurity, Astana IT University, Astana, Kazakhstan; ^3^Department of Biomedical Sciences, School of Medicine, Nazarbayev University, Astana, Kazakhstan

**Keywords:** meat consumption, Central Asia, 3D food printing, plant-based diets, protein-rich plants

## Abstract

3D food printing (3DFP) is emerging as a vital innovation in the food industry’s pursuit of sustainability. 3DFP has evolved to significantly impact food production, offering the capability to create customized, nutritionally balanced foods. Central Asia has a higher than global average level of meat consumption *per capita*, which might be influenced by its historical and cultural background of nomadism. This dietary trend might potentially result in negative impacts on both the environment and human health outcomes, as it leads to increased greenhouse gas emissions and increased risk of chronic diseases. Reducing meat consumption holds the potential to address these sustainability and health issues. A possible strategy to reduce meat consumption and promote plant-based foods is 3D Food Printing (3DFP), which can rely on plant-protein sources from the region to create appealing and tasty alternatives for these populations. This review summarizes recent studies on plant protein-rich materials for 3DFP as a substitute to meet the growing global demand for meat as well as the 3DFP printing parameters associated with the different plant-based proteins currently used (e.g., lentils, soybeans, peas, and buckwheat). The findings revealed that buckwheat, a dietary staple in Central Asia, can be a promising choice for 3DFP technology due to its widespread consumption in the region, gluten-free nature, and highly nutritious profile.

## 1 Introduction

In recent times, there has been a noticeable increase in the consumption of meat, contributed by the burgeoning global population and increased affluence in various regions (1) Average meat consumption increased by almost 60 percent across the world, while consumption *per capita* increased by almost 25 percent. Meat consumption is expected to continue growing by 1.7 percent per year through 2022 (2). Besides potential health problems caused by excessive meat consumption, there has been concerns raised about the environmental impact of meat production, contributing to issues such as increased greenhouse gas emissions, water depletion, pollution, and loss of biodiversity (3). A possible strategy to address the issues involves adopting a more plant-based diet with reduced reliance on meat consumption. This approach holds significant relevance, particularly in Central Asia (CA) (4) which consists of five countries, namely Kazakhstan, Uzbekistan, Kyrgyzstan, Tajikistan, and Turkmenistan.

The CA region is composed of diverse cultures, many of which originated from nomadic lifestyle and practices that depend on livestock farming and meat consumption as a main source of nourishment (5). In these societies, meat is not just a simple dietary choice; it has been an integral component of their way of life for many generations. Beyond providing essential nutrients, it sustains communities in remote terrains with adverse and harsh climatic conditions, preserving cultural identities (6). As the region undergoes rapid urbanization and dietary transformations, adapting these traditional dietary practices related to meat production and consumption presents unique challenges. Encouraging the population to shift toward diets richer in plant-based proteins poses practical challenges, necessitating innovative approaches. One potential solution is the application of 3D food printing (3DFP) to create meat analogs based on plant-based proteins, which are both acceptable and familiar to the populations in this region, offering a novel and sustainable dietary option.

**Figure 1:**
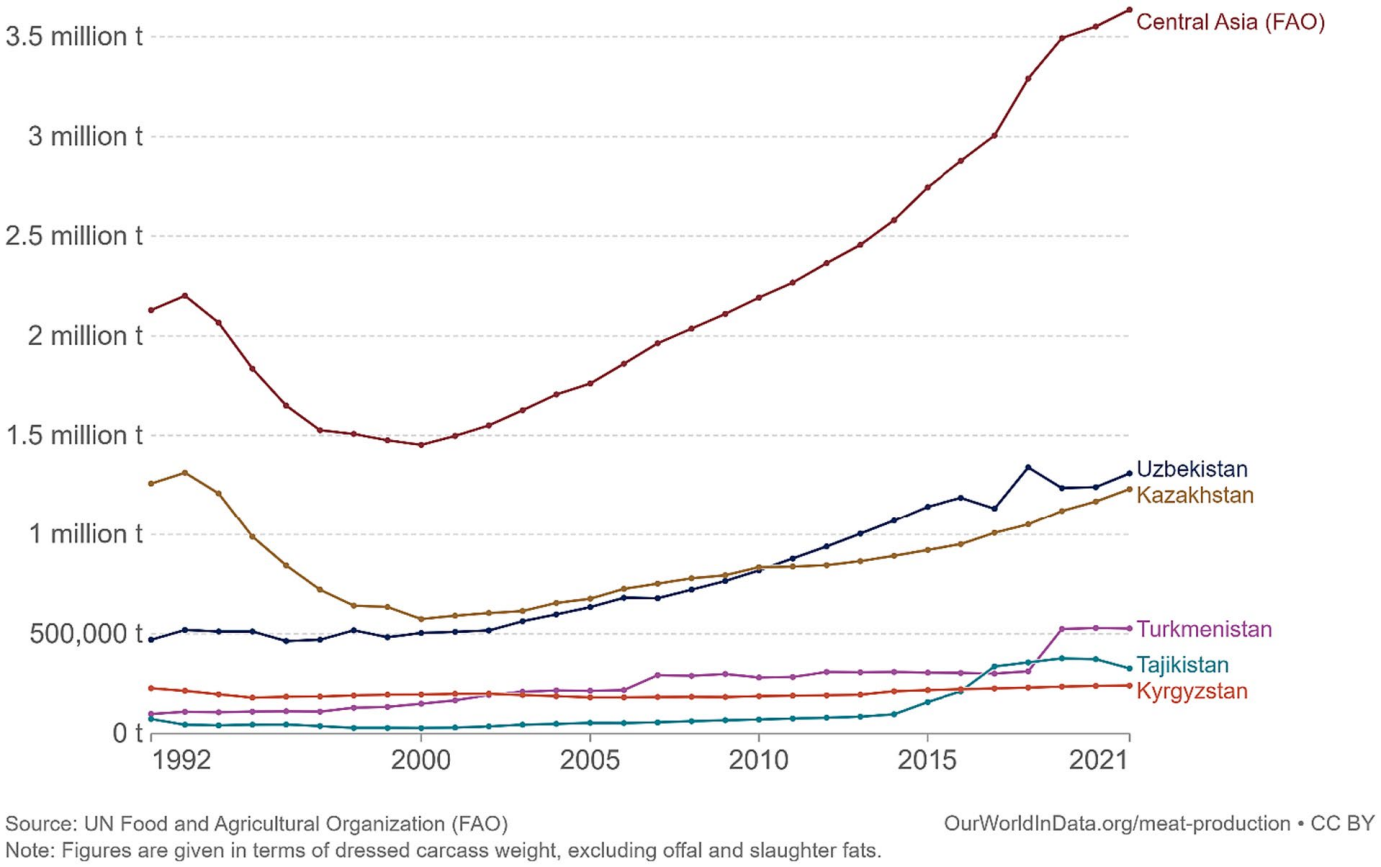
Meat production in Central Asia in the last decades (7).

According to the United Nation’s Food and Agricultural Organization (FAO), in CA meat production has been increasing dramatically since 2000s up to 2021 (Figure 1) (7). This trend of rapidly growing meat production and consumption could potentially yield negative consequences, including environmental degradation and adverse public health impact. Particularly, the possible adverse effects include the strain on global resources caused by the extensive water and land usage in livestock farming (5), as well as the health risks associated with excessive consumption of processed meats (8–13).

Consequently, promoting alternatives to meat protein consumption is a critical issue for both food security and public health.

The average meat consumption in CA ranges from 50 kg to 70 kg *per capita* per year. The average daily intake (124.76 g/day) for CA is reported to be one of the highest (14). According to World Population Review, Kazakhstan has the highest *per capita* consumption of sheep meat in the world, with an average of 8.5 kilograms per person annually. It has been recently shown that replacing a diet high in animal-based protein foods to one higher in plant-based protein foods can be beneficial for reducing risks factors associated with cardiovascular diseases and all-cause mortality (15, 16). Alternative plant-based diets are advantageous since not only are they rich in vital nutrients such as vitamins, minerals, and fiber, but they also have a lower environmental impact compared to meat-based diets. This potentially results in a substantial decrease in greenhouse gas emissions and the preservation of essential water and land resources, ultimately fostering an eco-friendlier food system (17). The structure of this paper is organized in such a way that it first discusses various strategies involving 3DFP for reducing meat consumption in *CA. It then explores the utilization of plant-based proteins like soybean, pea, lentil, and buckwheat for 3DFP.* Additionally, the review assesses how different printing parameters, such as print speed, layer height, and nozzle size, impact the quality of the printed food items and the properties of the plant-based materials used. Furthermore*, the unique properties of buckwheat in the context of 3DFP are considered, and* challenges *in* 3DFP’s *use within th*e food industry *are outlined*. Finally, the challenges in 3DFP’s use within the food industry are outlined with respect to use.

## 2 The need for sustainable food solutions

To reduce the CO_2_ emissions of food production, it is important to move from a meat-based diet to a fruits and vegetables diet. This will help achieve sustainability in food production.

### 2.1 Reducing meat consumption in CA

In regions similar to CA, where a long-standing tradition of high meat consumption prevails, promoting plant-based diets requires innovative strategies that should be informed by both scientific principles and cultural considerations (18). To be effective, these strategies must take into account various factors, including taste preferences, cultural norms, preferences for familiar sensory experiences, and the prevailing symbolism of having meat in the diet (6). The challenges of shifting dietary patterns in the CA region are amplified by the strong meat-eating culture, deeply rooted in nomadic traditions. Throughout history, meat has served as a fundamental source of sustenance, intimately intertwined with the way of life in the region. This profound cultural attachment to meat consumption continues to pose a significant barrier to the widespread acceptance of alternative dietary choices (15). Therefore, any efforts to promote plant-based diets in this context must be thoughtfully developed, considering both the scientific and cultural dynamics at play.

### 2.2 Environmental and health benefits of plant-based diets

To enhance the promotion of plant-based diets, an alternative strategy involves increasing the awareness of their health benefits (19). Extensive research demonstrates that adopting plant-based diets can significantly lower the risk of chronic diseases such as heart diseases, diabetes, colorectal, pancreatic and prostate cancer (20–22).

Emphasizing the environmental advantages of plant-based diets is another effective approach. By reducing meat consumption, individuals can actively participate in mitigating the significant impact of livestock farming on greenhouse gas emissions and deforestation, ultimately promoting a more sustainable future. (23–25).

Additionally, it may be helpful to introduce plant-based alternatives that are similar in taste and texture to traditional meat dishes (26). Educational campaigns can help raise awareness about the benefits of plant-based diets and provide information on the preparation of healthy and delicious plant-based meals (27).

**Figure 2:**
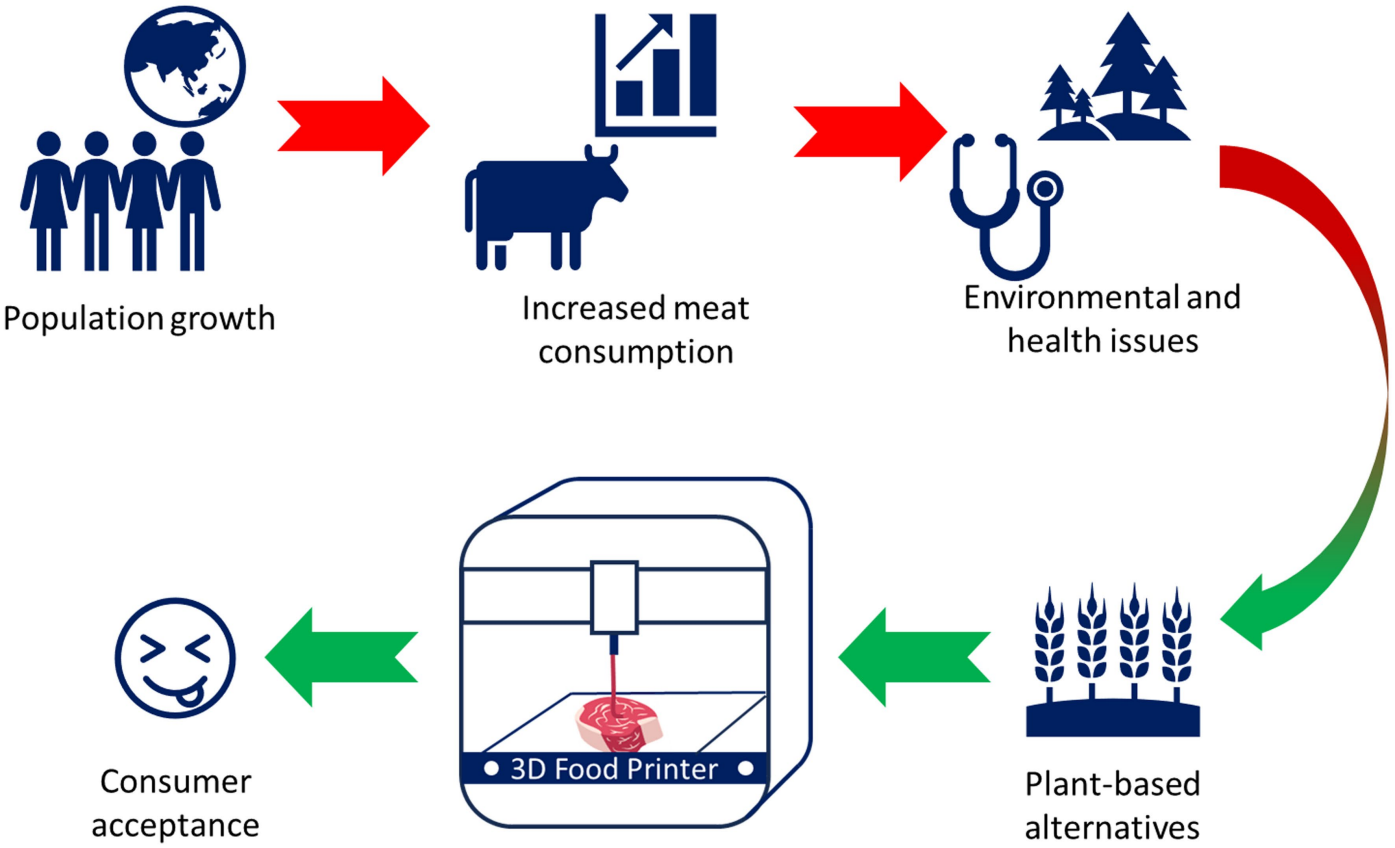
Towards reducing meat consumption by 3D food printing protein rich plants.

Emerging technologies such as 3DFP can provide exciting opportunities to influence dietary preferences and promote plant-based diets in CA by offering healthy, individualized substitutes for traditional meat consumption (28–30). Figure 2 provides a visual representation of the shift from a meat-based diet to a plant-based diet that is still rich in protein, a transition that is facilitated by the innovative use of 3DFP.

## 3 Potential of 3D printing in plant-based food production offering customization and innovation

3D printing technology has been gaining attention as an innovative approach to reducing meat consumption. The technology involves producing meat substitutes using plant-based ingredients that are 3D printed into the desired shape and texture (31). This approach has several potential benefits, including alleviating the environmental impact of meat production and improving animal welfare by reducing the need for livestock (10).

One of the most significant applications of 3D printing technology is the production of cell-based meat (32), which involves using animal cells to create meat products without the need for raising and slaughtering animals. The process involves the collection of a small sample of animal cells, which are consequently grown in a laboratory to produce muscle tissue. The resulting meat products are identical to traditional meat in terms of taste and texture offering customization to a greater extent and a substantially lower environmental impact (33).

While 3DFP technology is still in its early stages, it has the potential to revolutionize the food industry by providing a more sustainable and ethical alternative to traditional meat production. As research in this field progresses, an increase in innovative applications of 3D printing technology in the food industry can be expected.

## 4 Plant-based materials for 3D food printing

This section will discuss the different plant-based protein currently available and their applications in 3DFP.

### 4.1 Plant-based proteins

Plant-based materials, specifically proteins, are an excellent source of nutrition and a great alternative to meat proteins. Some popular plant-based protein sources include pea, buckwheat, soybean and lentil.

- Pea protein is a high-quality protein that is rich in essential amino acids, particularly lysine and threonine, (34). This protein is also easily digestible and has been shown to help reduce blood pressure (35) and improve kidney function (36).
- Buckwheat, on the other hand, is a gluten-free grain that is high in protein and fiber. It is also rich in antioxidants and has been shown to help lower cholesterol levels (37).
- Lentils, which are high in protein and fiber, may be ground into flour to make textured, protein-rich foods with customizable taste (38).
- Soybeans, also known for their high protein content, are employed in plant-based alternatives, giving sustainable possibilities for meat and dairy substitutes in 3D printing applications (39).

Incorporating these plant-based proteins into one’s diet can be a great way to improve health and reduce environmental impact. These plants can be used to prepare a variety of dishes, including soups, stews, salads, and smoothies, and to supplement a daily diet in the form of protein powder as well.

### 4.2 Applications of protein-rich plants to 3DFP

The use of 3D printing technology in food production has opened new possibilities for creating personalized nutrition and sustainable food system. Various plant-based food sources, such as soybean, pea, lentil, and buckwheat, offer diverse nutritional profiles that can be utilized for 3DFP. However, it is important to note that these protein-rich plants are most effective when combined with other ingredients and under optimized experimental conditions, as outlined in Table 1. By taking these factors into account, the potential for sustainable and nutrient-rich3D-printed food production can be fully realized.

**Table 1:** Application results for protein-rich plants in 3D food printing.

Crop type	Additional materials	Testing conditions	Optimum settings	Outcome observations	Reference
Soy	Xanthan gum (2%)	Mixing and heating in oil	Refrigerator: T: −80°C, 5 min; Oil, T: 170°C, 5 min	Textured soybean protein (TSP) and xanthan gum have almost identical texture properties as real chicken.	(31)
	Guar gum (0.05%), NaCl	Mixing	Mixing: SPI dispersion with soybean oil, 11,800 rpm, 5 min; Guar gum with SPI, 250 rpm, 1 min	Soy protein isolate (SPI) and guar gum (GG) inks exhibited minimal deviation in dimensional accuracy and excellent self-supporting properties.	(40)
	Sodium alginate (0.5 g), Gelatin (2, 6 g)	Mixing and storage	Mixing: magnetic stirring at 45°C; Storage: 4°C, 24 h	The addition of sodium alginate, gelatin, and soy protein isolate (SPI) paste resulted in 3D-printed foods with higher printing parameters like hardness, resilience, cohesiveness, springiness, and chewiness at higher temperatures.	(41)
Pea	Rapeseed oil (70%)	Mixing and homogenizing	Mixing: protein dispersion, 6,000 rpm, 15 s; rapeseed oil 10,000 rpm, 60 s; Homogenized: 650 bar	Printed objects with jammed emulsion based on pea protein particles could be easily extruded and retain their printed structure for 48 h.	(42)
	Pea protein hydrolysate (0–5%)	Mixing	pH: 8; Mixing: 20°C, 10 min	The addition of pea protein hydrolysate (PPH) to pea protein isolate (PPI) paste will result in better printability, and modified rheological properties of food ink even with high protein content.	(43)
	Xanthan gum (0–1%)	Mixing, heating, and storage	pH: 7.4; Mixing: 20°C; Heated: 92°C, 1 h; Stored: 4°C, 12 h;	3D-printed food based on pea protein isolate (PPI) in combination with 0.3% of xanthan gum could be used as a diet food for people with dysphagia.	(44)
	Potato starch (45%)	Mixing and drying	Drying: 38°C	The combination of pea protein and potato starch makes starch printable and enhances its cohesiveness and adhesiveness, and thermal properties	(45)
Lentil	–	Cooking and rinsing	Cooked 15 min in unsalted boiling water, dried, rinsed, blended	3D-printed food mixed with lentil demonstrated greater amount of iron.	(46)
	Casein powder	Mixing and cooling	Mixed with powder from bovine milk, refrigerated at 4°C	Lentil dough was produced with high-fiber, high-protein and low-fat characteristics.	(47)
Buckwheat	Deionized water (1:3 ratio)	Mixing and steaming	Mixing; Steamed at 1 atm pressure, 20 min	Among the other materials (rice, beam, etc.) has second best viscosity and viscoelasticity	(48)
	High-methoxy pertin (9:1 ratio) and deionized water (1:6 ratio)	Steaming	Steaming: 1 atm, 30 min until 90°C	Prepared samples showed more plastic behavior. In addition, decreased viscosity and flow point resulted easier extrusion through the nozzle.	(49)
	–	Stirring and cooling	Steered in water bath at 80°C, 17 min. Cooled until 20°C, refrigerated at 4°C	Buckwheat starch was used to print samples that showed good self-supporting properties.	(50)

Soybeans have long been a staple in foods made from plants due to their high protein content. Soy-based components such as soy protein isolates or soybean flours can be used in 3DFP to produce meat and dairy replacements as well as other plant-based goods (31). It is now possible to create textured and esthetically pleasing soy-based meals that imitate the qualities of conventional animal-based goods by printing complex structures with exact control over component ratios (51). For example, textured soybean protein (TSP) in combination with xanthan gum (2%) closely replicates the texture of real chicken (31). Additionally, excellent dimensional accuracy and great self-supporting properties can be achieved by soy protein isolate (SPI) and guar gum (0.05%) (40) by incorporating sodium alginate, gelatin, and SPI paste, 3D-printed soy-based foods can exhibit enhanced characteristics such as hardness, resilience, cohesiveness, springiness, and chewiness at higher temperatures (41). Furthermore, soybeans also have the additional advantage of being a sustainable source of protein, which fits with the expanding need for environmentally friendly food manufacturing techniques in the 3DFP sector (52).

Pea-based materials for 3D printing are a remarkable addition to the repertoire of sustainable and eco-conscious filament options. These filaments offer strong layer adhesion, ensuring that each print layer sticks well to the previous one, resulting in a structurally sound and reliable final product (42). Experiments have shown that pea protein particles mixed with 70% rapeseed oil can be easily extruded and maintain their structure for up to 48 h (42). It was reported that pea protein hydrolysate (PPH) with inclusion of pea protein isolate (PPI) paste reveals enhanced printability and modified rheological properties of food ink with a high protein content (43). Adding 0.3% xanthan gum to 3D-printed PPI-based food allows it to meet the dietary needs of individuals with dysphagia (44). Potato starch (45%) combined with pea protein makes the mixed starch printable and improves its cohesiveness, adhesiveness, and thermal properties (53). Moreover, the renewability of peas not only addresses concerns of resource depletion but also contributes to the economic viability and accessibility of pea-based materials (54).

Lentils are a great source of protein and fiber and can be turned into lentil flour, which makes them an excellent choice for 3D printing textured and protein-rich food items (45). Lentils can be used in 3D-printed food to enhance iron content by cooking for 15 min in unsalted boiling water (46). Additionally, mixing lentil flour with casein powder, exhibits lentil dough with high-fiber, high-protein, and low-fat characteristics, expanding the potential applications of lentils in 3D printing (47).

Buckwheat is a unique material in the realm of 3D printing due to its distinct characteristics. When used as a filament, it offers a range of properties that make it suitable for specific applications. Firstly, buckwheat, a gluten-free pseudo-cereal, possesses a rich nutritional profile. It is abundant in fiber, vitamin B6, magnesium, zinc, and other minerals (55). Moreover, buckwheat-based filaments can produce prints with a unique, slightly grainy texture. This can be desirable for certain applications where a natural, organic appearance is preferred. Due to its highly suitable rheological properties for 3D printing buckwheat performs as the best option as an alternative material among cereal grains (48). Particularly, mixing and steaming with buckwheat with deionized water at 1 atm for 20 min resulted in the samples with second-best viscosity and viscoelasticity. Additionally, increased plastic behavior was reported when buckwheat starch is utilized with high-methoxyl pectin and deionized water (in a 9:1 ratio) (49). This also decreased viscosity and flow point by making easier extrusion through the nozzle. Furthermore, 3D-printed samples displayed good self-supporting properties like in soybean achieved by stirring and cooling buckwheat-based materials (50). In section 4, application of buckwheat in 3D printing will be discussed.

Table 1 summarizes various plant-based materials utilized in 3DFP. Further research is warranted to optimize processing techniques and explore potential synergies with other food materials.

## 5 Buckwheat as a focus in 3DFP

Buckwheat is very popular in CA and has a relatively high content of protein. Therefore, it can be seen as an ideal candidate to produce eco-friendly 3D-printed food, as it is produced locally.

### 5.1 Buckwheat’s historical significance and applications in 3D printing

Buckwheat is cultivated in nearly every country that farms grains for local consumption. The significance of buckwheat as a crop is worth mentioning, especially in less fertile areas particularly, in colder and high-altitude regions of Asia. The origins of buckwheat cultivation can be traced back to inland Southeast Asia, approximately around 6,000 BCE. Afterward, this crop gradually spread to CA and Tibet, eventually reaching the Middle East and Europe by the 15th century 52. *Fagopyrum esculentum* (common buckwheat) and *F. tataricum* (Tartary buckwheat) are the main cultivated species of buckwheat which are believed to have originated in upland southwestern China, which were separate from the primary hubs of agricultural regions linked to the cultivation of rice and millet (56).

The incorporation of buckwheat in 3DFP offers several advantages. First, the nutritional composition of this crop can contribute to the development of customized and nutritious 3D-printed food products. Its gluten-free nature and unique flavor add distinctive taste profiles to the printed food (56). A study by S. Ji et al. investigated the rheological behavior of buckwheat-based pastes for extrusion-based 3D printing (50). The study found that varying the buckwheat content influenced the flow behavior and printability of the dough. Owing to its aromatic components, buckwheat has a unique flavor and scent. Jie Shi et al., utilized a sensory-directed flavor analysis approach to study the key odorants of tartary buckwheat for the first time (57). 49 aroma-active constituents with flavor dilution (FD) factors ranging from 1 to 2,187 were achieved through solvent-assisted flavor evaporation (SAFE) followed by an extraction of the volatile compounds of tartary buckwheat. Moreover, Malgorzata et al. provide an overview of the most recent developments concerning the sensory attributes, consumer choices, and the analysis of volatile compounds in both buckwheat and products made from buckwheat (58).

### 5.2 Buckwheat: an eco-friendly ingredient for sustainable food printing

Due to its many advantages, buckwheat emerges as a viable material for 3DFP, especially in regions where buckwheat is common such as CA (59–61). Small-scale farmers have social prospects owing to the production of buckwheat, particularly in areas with a shortage of arable land (62–64). It supports regional economies by providing a sustainable crop with minimal input needs (65). Buckwheat production is advantageous from an environmental standpoint as well (66, 67). The reduced chance of soil erosion and chemical discharge is due to fewer synthetic inputs needed (68). The quick growth cycle of buckwheat and its capacity to flourish in a range of environments combine with sustainable farming techniques, promoting soil health and biodiversity preservation (69).

While the integration of eco-friendly ingredients as in the case of buckwheat in 3DFP showcases the potential for sustainable food production, it also brings us to the forefront of technical challenges in 3DFP. Next, the critical aspects of 3D printing technology that directly affect the production of food will be examined.

## 6 Challenges and future directions

To guarantee the successful integration of 3D printing in food manufacturing, critical printing parameters must be adequately understood and controlled. Key parameters such as layer height, print speed, and nozzle size offer valuable insights into how they influence the final printed food (70, 71). A layer height, specifically, refers to the thickness of each individual layer that constitutes the final object and is a fundamental parameter that directly affects the resolution and surface finish of a printed part. The smaller layer heights result in finer details and smoother surfaces, but they also increase printing time (72). A print speed determines how quickly the printer’s nozzle moves while extruding material. It affects the overall printing time and can impact printed food’s quality. Higher print speeds may lead to reduced print quality due to less time for each layer to cool, potentially causing issues such as stringing or warping (73). A nozzle size also plays a critical role; smaller nozzles yield higher-resolution prints with detailed features, while larger nozzles enable faster printing but at a lower resolution (74). Understanding and effectively adjusting these key parameters in 3D printing processes is crucial for achieving high-quality, accurate, and reliable prints.

Beyond the technical aspects of printing, procurement of high-quality plant-based materials, ensuring taste and texture consistency, associated cost-effectiveness and scalability of 3DFP poses additional challenges for broad adoption (29). Consumer acceptance and awareness of 3D-printed foods could pose challenges due to individuals’ reluctance to include the 3D printing technology in their dietary habits (30). There is a need for strategic marketing and educational campaigns to build trust and familiarity with 3D-printed foods (3). The market trends indicate a potential shift toward innovative food technologies that align with sustainability and health trends (75), but this shift will require continuous engagement with consumers to understand their preferences and address their concerns. Therefore, overcoming the technical, economic, and consumer-related challenges might be a key to unlocking the full potential of 3D printing in reduced meat consumption.

## 7 Conclusion

CA’s escalated meat consumption poses significant threats to the environment and public health. Particularly, environmental degradation, freshwater pollution, and increased greenhouse gas emissions are the potential consequences of meat production. Thus, there is an urgent need for the promotion of sustainable diets that advocate for the reduction of meat consumption. This challenge can be addressed by utilizing plant-based alternatives which are essentially enriched with vitamins and ingredients that can lower environmental impact and provide a healthy lifestyle.

The convergence of 3D printing technology with the food industry holds significant promise for mitigating global meat consumption. Plant-based substitutes without relying on traditional livestock can be enabled by 3DFP In places such as CA where meat consumption is firmly ingrained in culture, 3DFP can play a critical role in manufacturing plant-based alternatives that mimic the flavor and feel of traditional meat, helping the transition to more sustainable eating patterns. Nevertheless, currently, the list of 3D food printable food types is still limited. Furthermore, the technology’s cost for professional use, coupled with the need to address consumer acceptance, should be taken into account, as adoption will require time.

## Author contributions

UA: Data curation, Investigation, Project administration, Writing – original draft, Writing – review & editing. AA: Data curation, Investigation, Writing – original draft, Writing – review & editing. TS: Data curation, Investigation, Writing – original draft, Writing – review & editing. DD: Data curation, Formal analysis, Investigation, Validation, Writing – review & editing. DT: Conceptualization, Supervision, Writing – review & editing. M-YC: Conceptualization, Data curation, Formal analysis, Investigation, Methodology, Project administration, Supervision, Writing – original draft, Writing – review & editing.
